# Interventions on Microbiota: Where Do We Stand on a Gut–Brain Link in Autism? A Systematic Review

**DOI:** 10.3390/nu14030462

**Published:** 2022-01-20

**Authors:** Margherita Prosperi, Elisa Santocchi, Letizia Guiducci, Jacopo Frinzi, Maria Aurora Morales, Raffaella Tancredi, Filippo Muratori, Sara Calderoni

**Affiliations:** 1 Department of Developmental Neuroscience, IRCCS Stella Maris Foundation, Viale del Tirreno 331, 56128 Calambrone, Italy; margherita.prosperi@fsm.unipi.it (M.P.); jacopo.frinzi@studio.unibo.it (J.F.); raffaella.tancredi@fsm.unipi.it (R.T.); filippo.muratori@fsm.unipi.it (F.M.); 2 UFSMIA Zona Valle del Serchio, Azienda USL Toscana Nord Ovest, 55032 Località Castelnuovo Garfagnana, Italy; santocchielisa@gmail.com; 3 Institute of Clinical Physiology, National Research Council, Via Moruzzi 1, 56124 Pisa, Italy; letiziag@ifc.cnr.it (L.G.); morales@ifc.cnr.it (M.A.M.); 4 Department of Clinical and Experimental Medicine, University of Pisa, Via Roma 55, 56126 Pisa, Italy

**Keywords:** probiotics, prebiotics, fecal microbiota transplantation, psychobiotics, microbiota, gastrointestinal, ASD

## Abstract

The alteration of the microbiota–gut–brain axis has been recently recognized as a critical modulator of neuropsychiatric health and a possible factor in the etiopathogenesis of autism spectrum disorders (ASD). This systematic review offers practitioners an overview of the potential therapeutic options to modify dysbiosis, GI symptoms, and ASD severity by modulating the microbiota–gut–brain axis in ASD, taking into consideration limits and benefits from current findings. Comprehensive searches of PubMed, Scopus, the Web of Science Core Collection, and EMBASE were performed from 2000 to 2021, crossing terms referred to ASD and treatments acting on the microbiota–gut–brain axis. A total of 1769 publications were identified, of which 19 articles met the inclusion criteria. Data were extracted independently by two reviewers using a preconstructed form. Despite the encouraging findings, considering the variability of the treatments, the samples size, the duration of treatment, and the tools used to evaluate the outcome of the examined trials, these results are still partial. They do not allow to establish a conclusive beneficial effect of probiotics and other interventions on the symptoms of ASD. In particular, the optimal species, subspecies, and dosages have yet to be identified. Considering the heterogeneity of ASD, double-blind, randomized, controlled trials and treatment tailored to ASD characteristics and host-microbiota are recommended.

## 1. Introduction

Autism spectrum disorders (ASD) are neurodevelopmental disorders characterized by persistent social communication difficulties with concurrent restricted interests, repetitive activities, and sensory abnormalities [1]. According to a recent Italian study, ASD have a prevalence of about one in 87 children aged between 7 and 9 years [2].

The high prevalence of some specific medical comorbidities, such as food selectivity and gastrointestinal (GI) disorders, in subjects with ASD compared to typical-development (TD) peers has led to a growing interest in organs and systems other than the central nervous system (CNS), but closely related to it. In recent years, research has focused on the role of bidirectional communication between the intestine and the brain (“the gut–brain axis”) in the etiopathogenesis of various stress-related psychopathological disturbances and neuropsychiatric conditions, including ASD, providing an essential contribution to understanding the diseases and proposing new therapeutic perspectives [3]. It has been hypothesized that the presence of alterations in the gut microbiota, a complex community of microorganisms living in the intestine and including anaerobic bacteria and viruses, protozoa, archaea, and fungi, could cause secondary effects at the level of the CNS [4].

Different authors have hypothesized that GI disorders and alterations in the gut microbiota could contribute to the expression of the autistic phenotype or exacerbate the severity of symptoms in subjects genetically predisposed to ASD [5,6,7,8]. As emerges in studies on animal models, the microbiota is essential for developing social relationships. By re-establishing a condition of eubiosis in the intestinal microbiota during a specific developmental time window in germ-free mice, or in the maternal immune activation mouse model of ASD, it is possible not only to correct the defects of permeability and intestinal dysbiosis but also to act on ASD symptoms by reducing the production and absorption of toxins in the intestine [9,10]. In pioneering research [11], oral administration of minimal doses of vancomycin was associated with significant improvements in children’s behavior with regressive ASD. However, treatment had time-limited beneficial effects that ceased when therapy stopped; on the other hand, antibiotic treatment was not justifiable for prolonged periods. Therefore, it has been hypothesized that oral antibiotic therapy with vancomycin could temporarily improve chronic dysbiosis [11], indirectly reducing the increased intestinal permeability and indirectly acting on behavioral symptoms typical of ASD.

On the basis of these findings, treatments acting at the gut microbiota level, such as prebiotics, probiotics, and fecal microbiota transplantation (FMT), promise a reduction in GI symptoms and autistic symptoms in individuals with ASD, as already partially shown by other researches [12].

Probiotics are nonpathogenic living microorganisms considered beneficial to human health when administered in adequate quantities as a dietary supplement. They have recently been defined as “psychobiotics” because they are considered a therapeutic tool, influencing brain development and behavior through their activity in restoring the healthy balance of the intestinal microbiota, producing and/or modulating the levels of neurotransmitters [13]. Commonly used probiotics are *Lactobacillus*, *Bifidobacterium*, *Saccharomyces cerevisiae*, and some *Escherichia coli* and *Bacillus* species.

Prebiotics are nondigestible substances naturally contained in some foods (such as resistant starch, nonstarch polysaccharides, oligosaccharides, galacto-oligosaccharides and xylo-oligosaccharides), which selectively stimulate the growth of probiotics such as *Lactobacilli* in the intestine and *Bifidobacteria* [14]. Promising results derive from the studies on prebiotics [15], although their administration on children with ASD is still in the initial experimentation phase.

The symbiotic treatments, a combination of probiotics with prebiotics, also resulted in a positive modulation of the gut microbiota and metabolic activity of children with ASD [16].

FMT or fecal bacteriotherapy is a nondrug medical treatment in which fecal material from a donor is treated in the laboratory and placed orally in the recipient as capsules, through endoscopic procedures (i.e., colonoscopy, orogastric tube) or with enema. FMT has been proposed as a popular treatment for refractory *Clostridium difficile* infection, obesity, chronic inflammatory bowel diseases, and recently as a therapeutic strategy for autism [17]. A study concerning the duration of a single transplant observed that there is a significant impact on the microbiota up to 24 weeks [18]. Few clinical studies have evaluated the impact of the FMT or microbiota transfer therapy (MTT, i.e., a modified FMT protocol) on autistic symptoms in individuals with ASD [19,20,21].

Here, the promising prospects deriving from the studies on probiotics, prebiotics, and FMT in subjects with ASD are summarized.

## 2. Materials and Methods

This systematic review was conducted in accordance with the Preferred Reporting Items for Systematic Reviews and Meta-Analyses (PRISMA) statement [22]. The research was conducted on several databases and search engines (PubMed, Scopus, Web of Science Core Collection, EMBASE) from 2000 (the date of the first publication on this topic) to December 2021, using the following string: “(autism OR ASD OR autism spectrum disorder OR autism spectrum disorders OR autistic) AND (pro-biotic OR probiotic OR probiotics OR probiotic therapy OR prebiotic OR prebiotics OR prebiotic therapy OR fecal microbiota transplantation OR microbiota transfer therapy)”.

All articles providing sufficient information about studies concerning therapeutic strategies focusing on the gut–brain axis in ASD were included.

The inclusion criteria were: (1) studies in humans; (2) a primary clinical diagnosis of ASD in the enrolled subjects; (3) the effects of probiotics, prebiotics, symbiotic treatments, and FMT as the main topic of the paper; (4) clinical studies (e.g., biological, biochemical, or molecular studies were excluded); (5) studies considering behavioral/symptomatic changes besides plasmatic/fecal effects after treatment (6) research articles only (reviews, meta-analyses, study protocols, case reports, conference abstracts, letters to the editor, commentary, preliminary study, or preprint were excluded); (7) English language only.

Two reviewers evaluated the extracted studies independently, applying the inclusion and exclusion criteria to minimize random errors and bias at all stages of the review process. If consensus could not be found, a third reviewer was included, and disagreements about whether an article should be included were resolved through discussion. Finally, a manual search of additional references on this subject was carried out to identify articles not included, also considering papers cited in previous reviews. All reviewers discussed the selected data and addressed the extracted data.

First, 1769 publications found through the database search and based on inclusion criteria were selected. Secondly, papers in duplicate, reviews, and papers other than research articles were removed by automation tools. Thirdly, the abstracts of each of the remaining articles (*n* = 357) were reviewed and selected based on the subject matter of the study, excluding nonhuman models/research models, papers examining other diseases or conditions, narrative reviews not identified in the previous screening, and preclinical and miscellaneous studies. Finally, 35 full texts, integrated them with four papers found on reading the references of other papers/reviews, were assessed for eligibility.

The methodological quality of the included studies was assessed independently by two reviewers. Randomized controlled trials (RCT) were evaluated using the revised Cochrane-risk of-bias tool for randomized trials (RoB 2) [23], which includes six domains of bias: bias arising from the randomization process, bias due to deviations from intended interventions, bias due to missing outcome data, bias in the measurement of the outcome, bias in the selection of the reported result, and overall bias. Nonrandomized studies were evaluated using the “risk of bias in nonrandomized studies-of interventions” (ROBINS-I) [24], which includes seven domains of bias: two included in the pre-intervention stage (bias due to confounding, bias in selection of participants into the study), one in at intervention stage (bias in the classification of interventions), and the last four in the postintervention stage (bias due to deviations from intended interventions, bias due to missing data, bias in the measurement of outcomes, and bias in the selection of the reported result). If at least one of the domains was rated as high, the trial was considered at a high risk of bias. If all domains were judged as low, the trial was considered at low risk of bias. Otherwise, the trial was considered as having an unclear risk of bias. Disagreements in scores of each of the domains were resolved through discussion between reviewers.

## 3. Results

**Figure 1:**
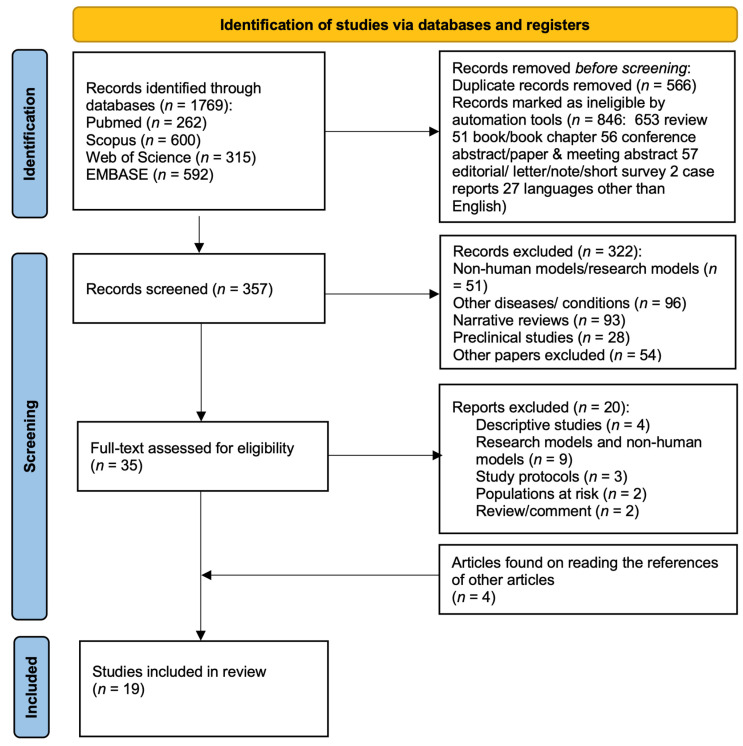
Flow diagram of studies evaluated in the systematic review based on the PRISMA 2020 statement. From: Page MJ, McKenzie JE, Bossuyt PM, Boutron I, Hoffmann TC, Mulrow CD, et al. The PRISMA 2020 statement: an updated guideline for reporting systematic reviews. *BMJ* 2021, 372. doi.org/10.1136/bmj.n71.

The flowchart for the PRISMA method used in this systematic review is shown in Figure 1.

A total of 19 papers we found to be suitable for this review. For an overview of the studies concerning the use of prebiotics, probiotics, symbiotics, and FMT in subjects with ASD published up to December 2021, see Table 1.

## 4. Study Characteristics

The majority of the studies (n 9) [11,25,26,27,28,29,30,31,32] deal with the use of probiotics in subjects with ASD, the other ones treat the use of prebiotics (4 studies) [33,34,35,36], and mixed probiotics–prebiotics therapy (3 studies) [37,38,39] in ASD, and only three [19,20,21] are about FMT. Studies were conducted in seven different countries: England (*n* = 2), U.S. (*n* = 6), Poland (*n* =1), Italy, (*n* = 2), China (*n* = 6), Egypt (*n* = 1), Japan (*n* = 1). Eight out of 19 studies are randomized controlled trials, possibly reducing certain sources of bias typical of studies measuring efficacies of an intervention. The subjects examined are predominantly males (603 males and 89 females with ASD; in two studies [27,30] the sex of participants are missing); all are children and adolescents between 1 and 17 years old with ASD described in studies published mainly in the last ten years. The sample sizes are relatively small, globally ranging from 11 to 85 subjects with a maximum of 131 subjects; in more than two-thirds of the studies, the reasons for the dropout/refusal to participate and any side effects of the treatment are clearly reported [11,19,21,25,28,29,30,31,32,33,37,38,39].

Most studies (16 out of 19) [11,19,20,21,25,26,27,28,30,31,32,33,35,37,38,39] reported GI symptoms as medical comorbidity associated with ASD. No study discusses the dietary habits or food intakes of enrolled subjects before or after the intervention (micronutrients and macronutrients in [33]), as well as interference/interaction with possible concomitant drug treatment, even if in two studies these data are collected [31,32] and in another two studies medications were excluded for at least one month [35], and antibiotics/probiotics in the last week [21] before the sampling period. Effective compliance with treatment by examining the fecal samples after the intervention or administering the treatment within a hospital setting is lacking; in a minority of cases [25,28,31,33,37,38], the compliance with treatment is verified through other ways (e.g., measured by packet counts of returned probiotic and placebo containers).

As reported in Table 1, six of the 19 analyzed studies did not present microbiota data, two used the fluorescence in situ hybridization (FISH) technique. In 10 studies, PCR was carried out, and the mixture of purified PCR products was generated for the next-generation sequencing (NGS) library.

The type of intervention varies across the trials, with wide variability in the strains and different formulations used (single-strain probiotic therapy [25,26,29,38], blended probiotic formulations [11,27,28,30,31,37,39], or both [32]) in studies examining probiotics. These interventions are primarily *Bifidobacterium* and *Lactobacillus* genus-based, with a minority of studies testing *Streptococcus* species; the most tested strains are *Bifidobacterium longum* and *infantis* (*n* = 4, both) and *Lactobacillus acidophilus* (*n* = 6). The therapy is mainly taken by mouth, in capsule or packet to be taken daily (once, two or three times/day), with doses ranging from 0.5 to 90 × 10^9^ CFU of bacteria. Treatment duration varied between 21 days and 6 months, with only three studies reporting postintervention follow-up outcomes [11,20,33].

The changes recorded in GI symptoms and ASD severity in the subjects examined were mainly obtained through tools administered to parents rather than a direct examination by the clinicians.

The most frequently used assessment instruments for ASD-related behavior are the ATEC [40] (*n* = 5) and ABC [41] (*n* = 8). In a minority of studies, specific instruments to directly assess the symptoms of autism and clinical severity are used (e.g., standardized assessments like CARS, ADOS, ADI, CGI). For the assessment of GI symptoms, the most used tool is the 6-GSI [6] (*n* = 3), a modified version of the GSI that has been designed for the ASD population. Other tools used are PedsQL GI Module Scales (*n* = 1), questionnaire on pediatric gastrointestinal symptoms–Rome III [42] (*n* = 1), GI History survey [43] (*n* = 1), and GI symptom rating scale [44] (*n* = 2; GSRS). Additionally, seven studies collect data on GI function with either unspecified questionnaires or qualitative GI diary.

Only 4 out of 19 studies (21.05%) include a healthy control group, with 1 for probiotic intervention [30], 1 for prebiotic treatment [34], 1 for both probiotic plus prebiotic therapy [37], and 1 for FMT [21]. Similarly, only 6 out of the 19 studies (31.58%) include a placebo group in the study design.

## 5. Study Quality

Unlike other reviews on this topic [45,46,47], the most appropriate tools to judge each paper were chosen, differentiating how to estimate biases of RCT from those of nonrandomized trials. Applying the tools adopted to specifically evaluate the risk of bias in RCT also in nonrandomized studies could represent a mistake [48], e.g., randomization protects against biases that arise before the start of intervention [24], then this phase has to be considered as a possible source of biases in not-randomized trials only. Some authors [49] evaluated studies quality with early versions of validated tools as the MINORS scale [50], or they simply did not value it [51]. Still, it has already been described how this is crucial in systematic reviews [52].

As shown in Table 2 and Table 3, most studies have a poor-quality design, with different concerns for bias.

As expected, RCT have a lower risk for bias than nonrandomized studies, but only two papers [29,31] reach a low overall risk of bias. All examined RCT showed a good randomization process and almost all a low bias due to deviations from intended interventions, measurement of the outcome, and selection of the reported result. The most problematic area is represented by bias related to the missing outcome data, in most cases as a consequence of the dropout of many of the participants, which leads to biased results. This is often not reported as a limit, resulting in a high risk-of-bias judgment.

In nonrandomized studies, the most critical methodological areas for the reviewed studies are represented by the pre-intervention and postintervention phases. Severe concerns were found, especially regarding possible bias due to deviations from intended interventions and selection of the reported results.

## 6. Relevant Data Emerging from Studies on Treatments Acting on the Gut–Brain Axis in ASD

### 6.1. Clinical Studies on Probiotics

By exploring the possible applications of probiotic therapy, not only case reports [53,54,55] have identified a beneficial effect of specific probiotics on some of the behavioral characteristics specific to, or associated with, ASD in small populations [11,25,26,27,28,29,30,37,38,39], or larger samples [31,32]. Moreover, GI symptoms such as constipation, stool consistency, flatulence, and abdominal pain were improved [27,28,30,31,37,38,39].

In the first double-blind placebo-controlled study published on this topic [25], the effects of supplementation with *L. plantarum WCSF1* were examined. In addition to a modulation of the intestinal microbiota, an improvement in stool consistency and behavioral and emotional problems in subjects with intellectual disabilities and global developmental delay were observed after administering the probiotic. However, the study’s statistical power was affected by a high drop-out rate.

In a cohort study [26] where oral supplementation of a strain of *L. acidophilus* was tested, there was an improvement in the ability of concentration and carrying out orders. From baseline, no difference in reacting to other people’s emotions or using eye contact was present. While intriguing, the small sample and open-label design of the study limit the relevance of the results. It is also of relevance that the total duration of the trial was double that of the intervention conventionally selected in clinical trials using probiotics on ASD [56].

In another uncontrolled clinical study [27], positive effects were recorded due to administering a mixture of five probiotic strains formulated with the immunomodulator Del-Immune V^®^ (*L. rhamnosus V* lysate) in a population of 33 children with autism and concomitant GI symptoms. In addition to reporting an improvement in GI symptoms, a significant improvement in autism severity was reported after only three weeks of treatment. Similar results emerge from a subsequent uncontrolled study on 30 children with ASD receiving three months of treatment with a probiotic mixture (strains of the species *L. acidophilus*, *L. rhamnosus*, and *B. longum*) and dried carrot [37]: the authors found significant changes in autism severity with concomitant improvement in gastrointestinal problems. These studies were characterized by limited sample size and an open-label design, with the consequent risk of overestimating the effects of probiotic therapy.

More limited results regarding probiotics in ASD emerge in a recent placebo-controlled study [29]: the authors highlighted how supplementation with *L. plantarum PS128* resulted in a reduction in anxiety, hyperactivity, and opposition/challenge behaviors, although without changes in ASD symptoms. Also, the stratified analysis by age allowed the authors to identify better effects on younger than older children, underscoring the importance of early interventions. It is also to note that almost all the scores evaluating the existing impairments decreased in the placebo group at week four, suggesting that the placebo effect and confounding factors may affect the results. The importance of a placebo-controlled study design for this type of investigation is therefore further emphasized.

In a randomized, double-blind, placebo-controlled study recently conducted [31], the effects of a six-month treatment with a mixture of probiotics (De Simone formulation) on 85 preschool children with ASD were evaluated. The De Simone formulation is a food supplement with a high concentration of bacteria (450 billion per sachet and 112 billion per capsule) containing eight different strains of bacteria (*S. thermophilus DSM 24731*, *B. short DSM 24732*, *B. longum DSM 24736*, *B infantis DSM 24737*, *L. acidophilus DSM 24735*, *L. plantarum DSM 24730*, *L. paracasei DSM 24733*, *L. delbrueckii subsp. bulgaricus DSM 24734*). There was no significant difference between the group that received probiotic treatment and the placebo group in the severity of autistic symptoms as assessed by the gold-standard scale for autism, the autism diagnostic observation schedule (ADOS)–2 [57]. However, a secondary exploratory analysis revealed a significant reduction in ADOS scores in children without GI symptoms treated with probiotics compared to the group receiving the placebo. In addition, significant improvements in some GI symptoms, adaptive functioning, and sensory profile were found in the probiotic-treated group of GI symptoms compared to the placebo-treated GI group.

Although this study has the most prolonged duration (6 months) than those already published on the effects of probiotics, the results must be interpreted with caution for the significant dropout rates (22/85; 25.9%), especially in the GI group.

In Niu et al. [30], the authors compared a group treated with a combined intervention with probiotics (a probiotic formulation of three *Lactobacillus* and three *Bifidobacteria* strains) and behavioral therapy to a group treated with behavioral therapy only. They started from an initial sample of 114 ASD subjects in which the fecal microbiota was profiled. Still, the final subgroups were smaller, comparing 37 subjects treated with probiotics and applied behavioral analysis (ABA) therapy (divided into GI and NGI subjects) with 28 subjects treated with ABA intervention only. Despite the encouraging results in the group treated with probiotics both in ASD symptoms and GI problems, there are different concerns of bias primarily related to the study design (unblinded study) where caregivers evaluated the gain, and the duration was somewhat limited (one month).

Similar results were found in a randomized, double-blind placebo-controlled study [39] using a formulation of four different probiotic strains (two of both *Lactobacillus* and *Bifidobacteria*) mixed with a prebiotic (fructo-oligosaccharide). However, the sample was relatively small, and the duration was not specified. Moreover, whether the effects are due only to the probiotic therapy or to the prebiotics/probiotics substrate mixture cannot be determined: a probiotic-only treatment group is missing, useful in parsing out specific treatment effects.

Recently, an open-label trial testing differences between *L. plantarum* (“the *L. plantarum* group”) and other probiotics (“the OP group”) in the treatment of GI and psychiatric symptoms was published [32]. The authors found that the positive effects were more evident in younger children, and the patients taking *Lactobacillus plantarum PS128* had more significant improvements and fewer side effects than the OP group.

Although such a large sample differentiates it from all other published works on this subject, several possible sources of bias limit the relevance of the results as the unblinded study design, the unbalanced number of subjects between groups, the heterogeneous treatment in the group testing other probiotics different from *L. plantarum*, and the lack of the microbiota analysis before and after treatment. As in other studies, the lack of information about microbiota changes during the treatment could limit the possible resulting correlations between the brain, clinical improvement, and specific microbiota composition in ASD.

Other studies concerning the administration of probiotics in subjects with ASD did not analyze the effects on the behavior of the enrolled subjects [6,58] (not included in this review) or did not reveal any improvement related to treatment with supplements [28,38].

In the randomized, double-blind trial by Sanctuary et al. [38], the treatment included five weeks of probiotic (*B. infantis*) and prebiotic (bovine colostrum prebiotic oligosaccharides) supplementation, followed by a two-week wash-out period and finally five weeks of supplementation only with prebiotic. Supplementation with the combined treatment did not seem to lead to a significant improvement in irritability and stereotyped behaviors, a result that instead emerged from the treatment with the prebiotic alone. Limitations include the small sample with a very high dropout rate (8/20 completed the study) and the lack of a probiotic-only treatment group, making it very similar to the study by Wang et al. [39]

In the placebo-controlled pilot study by Arnold et al. [28] about the use of a probiotic containing eight different bacterial species (mainly *Lactobacilli* and *Bifidobacteria*) in a sample of 13 children with ASD aged 3 to 12 years, no significant differences emerged as far as the improvement of the quality of life or the reduction of anxiety symptoms. Still, significant improvements in GI discomfort were seen during probiotic treatment compared to the placebo treatment period. It should be noted the small sample size, with a high dropout rate (10/13 completed the study), and the use of a new anxiety scale, not necessarily sensitive to highlight possible clinical changes.

### 6.2. Clinical Studies on Prebiotics

Seven studies have been published to date that used different types of prebiotic compounds, including carrot powder [37], partially hydrolyzed guar gum [35], vitamin A [34,36], and galacto- [33,38] and fructo-oligosaccharides [39]. Some authors have examined their effects when administered alone [33,34,35,36] or associated with probiotics [37,38,39]. Therefore, in the latter studies, it is not possible to determine whether the effects/benefits are due to the specific prebiotic or its function as a substrate for some probiotic strains.

In the paper by Inoue et al. [35], a significant decrease in microbial alpha-diversity and some cytokines and chemokines (IL-1, IL-6, and TNF-α) were highlighted in a small sample of constipated ASD children after administering a prebiotic diet based on guar gum and β-endoglucanase produced by an Aspergillus strain. Prebiotic supplementation also increased the frequency of bowel movements with a consequent higher frequency of defecations per week. The authors hypothesized that the improvements of the gut dysbiosis and constipation symptoms could, in turn, help attenuate the level of serum cytokines and behavioral irritability. However, the lack of a control group and of a blinded trial, as well as the missing information about diets pre- and postintervention, have an impact on the relevance of the results.

Grimaldi and colleagues [33] found a significant increase in the *Lachnospiraceae* family and significant changes in the fecal and urinary metabolites and antisocial behavior of 30 children with ASD after a prebiotic intervention with supplementation for six weeks with Bimuno galacto-oligosaccharides (B-GOS^®^: 80% galacto-oligosaccharides). Despite the study’s strength, which also considers the participants’ dietary habits assessed by 4-day food diaries as macronutrients and micronutrients intakes, there was a high dropout rate (63% completed the study). This could further limit the power of the results from an already relatively small sample (41 enrolled subjects).

Two pilot studies of the same research group [34,36] tested vitamin A supplementation in a sample of children with ASD, showing in one case [36] a significant increase in the *Bacteroidetes*/*Firmicutes* ratio without changes in autism severity and behavioral problems, while in the other [34] a reduction in the severity of autism and in serum levels of 5-hydroxytryptamine, which correlated positively with autistic symptoms. The somewhat conflicting results on the severity of autism and the lack of a placebo-controlled study design in both types of research limit these findings’ strength.

In conclusion, in terms of emotional–behavioral symptoms and related to ASD, some authors have found an improvement after the administration of prebiotics [33,34,35,38]. In contrast, others have not shown a pre–post treatment change [36] in ASD subjects. The variability in the choice of prebiotics, the simultaneous administration with probiotic strains, and the few studies published to date do not allow us to draw definitive conclusions about their benefits in subjects with ASD.

### 6.3. Clinical Studies on Fecal Microbiota Transplantation

The first open-label study on MTT evaluated the impact of this technique on a sample of 18 autistic children aged 7 to 16 years with moderate-to-severe GI symptoms [19]. An approximately 80% reduction in GI symptoms (significant improvement in constipation, diarrhea, abdominal pain, digestive problems) and symptoms related to autism were identified. The improvement persisted after eight weeks since the end of the treatment. The protocol included preliminary therapy with antibiotics for two weeks, intestinal washing, and maintenance treatment with antacid drugs. Laboratory investigations revealed partial engraftment of the donor’s microbiota with consequent benefits at the level of the intestinal microenvironment (increase in *Bifidobacteria*, *Prevotella*, *Desulfivibrio*). In one case, there was an adverse dermatological reaction to vancomycin, and in 12 subjects, an increase in hyperactivity and aggression up to three days after the end of the treatment. Despite the relevance of side effects and the complex implant procedure, the authors suggested the superiority of MTT over probiotic therapy due to the greater probability of engraftment as well as the presence of richer bacterial populations.

The authors then carried out a check on the same group of patients two years after the previous study [20], finding maintenance over time of both GI and autistic symptoms improvements and persistence of the increase of *Bifidobacteria* and *Prevotella* in the microbiota.

Recently, Li et al. [21] conducted an open-label study evaluating the effect of FMT on GI and ASD symptoms, as well as on gut microbiota alterations in children with ASD. To further strengthen their results, the authors also examined a TD control group, and found that FMT could shift the bacterial community of children with ASD toward that of their TD peers. Considering ASD children, FMT improved GI symptoms and ASD symptoms, with some effects persisting 8 weeks after treatment.

Although these results are promising, it should be emphasized that FMT and its applications are still in an experimental stage. Relevant side effects due to preventive treatment with antibiotics to favor the engraftment of the donor’s microbiota in the recipient [19], and other adverse reactions, including hyperactivity, aggressive behaviors, fever, and major changes in blood chemistry [21] were described.

## 7. Discussion

ASD can be considered a relatively frequent disorder with a high longitudinal diagnostic stability [59], characterized by a significant individual, familial, and societal burden [60]. To date, evidence-based rehabilitative interventions can improve global outcome for some ASD people [61], but the possibility of boosting these with an easily administered supplementary treatment that acts on the gut–brain axis, with limited side effects and low costs, should be adequately explored. As shown, the studies published to date that have examined integrative treatment with probiotics and/or prebiotics, and FMT are few and show heterogeneous results. Most authors, however, found a benefit of these therapies not only on GI disorders but also on behavioral problems and severity of autism symptoms, both in RCT and in nonrandomized studies. As already highlighted [62], not all these positive results reach statistical significance. Moreover, it is unclear if the period of supplementation that each study considers is long enough to expect behavioral changes.

Although it should be noted that there is a good level of truthfulness from parents about their child’s emotional and behavioral problems [63], the placebo effect should be considered when the improvement has been rated through questionnaires [64,65]. Moreover, a placebo group is lacking in many of the cited studies examining intervention on microbiota in ASD.

In general, significant limitations are the relatively limited study samples, frequently characterized by considerable dropout rates, participants that are exclusively children and adolescents, and study designs that often are unblinded trials. In addition, immediate or short-term effects are examined, while few studies analyze whether the benefits are maintained even at follow-up [11,20,33]. Considering FMT studies, some side effects may occur during the engraftment procedure of the microbiota and can be severe, requiring a suspension of the therapy [19] with side effects over time unknown.

As emerges from the methodological quality assessment of the 19 reviewed studies, only two [29,31] are RCT with an overall low risk of bias. It is of note that both studies are characterized by a relevant dropout rate (26% in Santocchi et al., 11% in Liu et al.) and that there are other possible sources of bias, such as lack of registration of participants’ dietary intakes and missing examination of comorbidities associated with ASD. Moreover, neither of the two studies analyzed the pre–post intervention changes in the microbiota and its relationship with the recorded changes in behavior. Therefore, it is not possible to establish whether biological changes in the gut mediate changes in behavior. Of note, only 10 of the 19 reviewed studies analyzed the microbiota with NGS. This method has simplified and improved the sequencing strategy, reducing the artifacts and cost of sequencing, as well as increasing the speed at which a genome can be sequenced, with differences across the available NGS platforms commonly used for sequencing [66].

Interestingly, the two studies showed relevant results in two different age ranges of participants: Santocchi et al. [31] in preschoolers, while Liu et al. [29] in school-aged children and adolescents with ASD. Considering previous results [29,32], where the ages of participants were relevant to expectations of more or fewer changes, further studies are needed to understand the exact correspondence between the type of treatment chosen and the available benefit by age group. In both studies, the authors comprehensively assessed ASD symptoms using clinical assessment tools and caregiver questionnaires, and this way of proceeding is also desirable for future studies examining this topic.

Beyond the type and duration of treatment, probiotics positively modify the fecal microbiota. A reduction of *Clostridia*; an increase in *Lactobacilli, Enterococci*, and *Bifidobacteria*; a normalization of the *Bacteroidetes/Firmicutes* ratio; a reduction in *Candida*, as well as a decrease in intestinal inflammation and permeability in children with ASD have been shown [25,26,28,37,58].

As emerges from this review, the consensus about types and doses of probiotics to be administered in ASD is lacking, ranging from single-strain to multistrain probiotics. Considering RCT studies examining the effects of probiotics exclusively, the most promising seem to be *L. plantarum WCFS1* [25], *L. plantarum PS128* [29], Visbiome [28], or Vivomixx [31] corresponding to the De Simone formulation, with positive results either in GI problems, severity of autism, or psychiatric symptoms. Unfortunately, long-term benefits are unknown because in none of these studies postintervention follow-up outcomes are conducted, hypothesizing temporary effects lasting only as long as the probiotic was administered.

Considering GI symptoms, it has been suggested that practitioners may consider probiotic therapy in children with ASD and severe GI dysfunction (e.g., constipation or diarrhea) because they may experience some reduction in symptoms [62]. Encouraging results, especially in contrasting GI symptoms, also emerge in studies analyzing prebiotics [33,35,38]. However, only one RCT study has been published to date [33] examining the role of the prebiotic Bimuno galacto-oligosaccharide without the confounding bias resulting from the simultaneous use of probiotics. Results indicated that this prebiotic modulated the gut microbiota composition, improved the anti-sociability scores when combined with an exclusion diet, but did not significantly affect GI symptoms. Therefore, the role of prebiotics must be deepened whether used alone or as a substrate for certain probiotic strains.

Moreover, considering the heterogeneity of ASD and that medications are specific for specific ASD subgroups only, as suggested by results of pharmacological trials in this population [67,68], none of the studies examining probiotics and/or prebiotics select the participants based on their intestinal microbiota—limiting possible positive results.

Much more needs to be done in research examining FMT in subjects with ASD. Despite the encouraging results, also considering long-term lasting after the end of the treatment [20,21], this area of research is in its infancy.

To date, no RCT studies are available on this topic, and the preparation procedure of its applications (as MTT) that require antibiotics, antiacid drugs, and bowel cleanse could be complex and not free from concerns, particularly when applied in children and adolescents with ASD. Moreover, as already shown [11,69,70], the effects of the preparation on the microbiota and its role in the benefits for the host beyond the microbiota transplant [19] must be considered. Regarding the microbiota changes in studies examining FMT in ASD subjects, Kang et al. have shown an increase of overall bacterial diversity and relative abundances of *Bifidobacteria, Prevotella, and Desulfovibrio* among other taxa, most of which persisted two years after the end of treatment [20]; whereas Li et al. [21] have observed that FMT could promote the colonization of donor microbes and shift the bacterial community of children with ASD toward that of the TD controls.

## 8. Conclusions and Future Perspectives

Despite the discrepant results between various studies, a different intestinal microbiota in individuals with ASD than in individuals with TD emerges. Although it remains to be clarified whether the above identified microbiota alterations are implicated in the onset of ASD or occur subsequently, there is growing evidence that they can aggravate autistic symptoms. It could happen either through a mechanism mediated by their action on the GI system or through indirect pathways related to the microbiota–gut–brain axis.

In recent years, several studies that have examined therapies acting on the microbiota–gut–brain axis as prebiotics, probiotics and FMT, have shown improvements in some gastrointestinal symptoms and some psychiatric symptoms in subjects with ASD. It should be noted that these treatments are easily administered, with limited side effects and low costs.

However, considering the variability of the treatments, the samples size, the duration of treatment and the tools used to evaluate the outcome, these results are still partial and do not allow us to establish a conclusive beneficial effect of probiotics and other interventions on the symptoms of ASD [62]. In particular, the optimal species, subspecies, and dosages have yet to be identified. Considering ASD heterogeneity, it would be desirable that treatments should be selected on the basis of the specific characteristics of both subjects with ASD and the host’s microbiota, with the ultimate goal to individualize the therapy [62].

## Figures and Tables

**Table 1:** Studies concerning the use of prebiotics, probiotics, and fecal microbiota transplantation in subjects with ASD; published until December 2021.

Reference	Country	Population	Intervention		Dose	Study Design	Microbiota Analysis (Sequencing Methods)	Main Results	Limits
Sandler et al. (2000)	U.S.	11 ASD (regressive onset) 10 ♂ 1 ♀ Age 3.5–7 yrs	PRO	Vancomycin + PRO (Lact acidophilus, Lact bulgaricus, Bifid bifidum)	Vancomycin (500 mg/d) 3/day × 8 wks, PRO (40 × 10^9^ CFU/mL) × 4 wks	Open-label trial	NO	Short-term improvement in ASD symptoms (CARS) during vancomycin treatment	Reduced compliance during PRO treatment No control group (microbiota compared with microbiota of adults) Small sample, unblinded study
Parracho et al. (2010)	England	62 ASD 59 ♂ 3 ♀ age 4–16 yrs	PRO	Lact plantarum WCFS1	4.5 × 10^10^ CFU/cp, 1 cp/day 3 wks per arm (PRO-wash out-PLA-wash out)	Randomized double blind placebo-controlled trial, cross-over	YES (FISH)	More aggressive and antisocial behaviors, anxiety problems and communication difficulties in the PLA group Improvement of the anti-social behaviors, anxiety, and communication problems No major differences in GI symptoms ↑ Lact/Enterococci and ↓ Clostridium coccoides found in the stools of ASD children as compared with PLA	Very high dropout rates (17/62 completed the study, 9 PRO and 8 PLA) No TD control group
Kaluzna-Czaplinska e Blaszczyk (2012)	Poland	22 ASD 20 ♂ 2 ♀ age 4–10 yrs Severe GI symptoms	PRO	Lact acidophilus (Rosell-11 species)	5 × 10^9^ CFU/g^2^/day × 2 mths	Open-label trial with self-control study	NO	Improvement in ability of concentration and carrying out orders; no difference in reacting to other people’s emotions or using eye contact	High risk of selection bias Unblinded study Microbiota not analyzed No TD control group No PLA group
West et al. (2013)	U.S.	33 ASD ♂♀missing age 3–16 yrs	PRO	DelPRO (Lact acidophilus, casei, delbrueckii + Bifid longum, bifidum + 8 mg Lact rhamnosus V lysate)	1 × 10^8^ billion CFU 3 times/day × 21 days	Open-label trial	NO	88% subjects ↓ ATEC total score, 48% ↓ diarrhea, 52% ↓ constipation	Risk of selection bias 25/33 reported ATEC scores, 21/33 returned stool frequency diaries Unblinded study Microbiota not analyzed No TD control group No PLA group
Kang et al. (2017)	U.S.	18 ASD with GI symptoms (moderate/severe) 16 ♂ 2 ♀ age 7–16 yrs	FMT	SHGM orally or rectally	Initial dose 2.5 × 10^12^ cells/day and maintenance dose 2.5 × 10^9^ cells/day for 7 or 8 weeks (+vancomycin + MoviPrep + Prilosec) Duration: 4 mths and 2 wks	Open-label trial	YES (NGS)	↓ 80% reduction of GI symptoms at the end of treatment lasting 8 wks after treatment. Behavioural symptoms of ASD significantly improved and continued improving 8 wks after treatment. ↑ diversity and abundance of Bifid, Prevotella and Desulfovibrio, increased after MTT, lasting for 8 wks	ASD symptoms changes not reported Small sample, unblinded study No TD control group No PLA group
Liu et al. (2017)	China	64 ASD 55 ♂ 9 ♀ age 1–8 yrs	PRE	20 study participants (17 ♂ 3 ♀) with plasma retinol deficiency (<1.05 μmol/L) treated with VA	200,000 UI once × 6 mths	Single-blind, nonrandomized, interventional pilot study	YES (NGS)	Significant ↑ Bacteroidetes/Firmicutes and ↓ Bifid; no change in the ASD severity or behavioral problems	No PLA group No TD control group
Grimaldi et al. (2018)	England	41 ASD 31 ♂ 10 ♀ age 4–11 yrs	PRE	Bimuno galacto-oligosaccharide (B-GOS^®^: 80% galacto-oligosaccharides)	1.8 g in powder (unknown frequency) × 6 mths. At the end of intervention, patients were followed-up for 2 additional weeks.	Randomized double blind placebo-controlled trial	YES (FISH)	Improvements in anti-social behaviour After treatment: ↑ Lachnospiraceae, significant changes in faecal and urinary metabolites	High dropout rates (26/41 completed the study) No TD control group
Guo et al. (2018)	China	33 ASD (28 ♂ 5 ♀) age 5.14 ± 1.33 yrs 32 TD; age 5.18 ± 0.87 yrs	PRE	VA in the 33 ASD	Single administration 200,000 UI	Open-label, interventional pilot study	NO	6 mths after administration: reduction of ASD severity and 5-hydroxytryptamine (positively correlated with ASD symptoms)	Unblinded study Microbiota not analyzed No PLA group
Shaaban et al. (2018)	Egypt	30 ASD 19 ♂ 11 ♀ age 5–9 yrs 30 HC children (relatives) age 5–9 yrs	PRE + PRO	Lact acidophilus + Lact rhamnosus +Bifid longum and dried carrot	1 g = 100 × 10^6^ CFU for each species 5 g/day × 3 mths	Open-label, prospective study	Unclear	↑ fecal levels of Bifid and Lact, significant improvements in ASD severity (↓ ATEC) and GI symptoms (6-GSI)	Unblinded study No TD control group
Arnold et al. (2019)	U.S.	13 ASD-GI-anxiety 6 ♂ 4 ♀ 6 ASD with PRO 4 ASD with PLA age 2–11 yrs	PRO	VISBIOME: 4 Lact strains (casei, plantarum, acidophilus, delbrueckii subsp Bulgaricus) + 3 Bifid strains (longum, infantis, breve)+ 1 Strept thermophilus strain and starch	9 × 10^5^ bacteria in half packet Half packet/2 times per day in the first 4 wks 1 packet/ 2 times per day if no effects are observed at 4 wks and 15 wks Duration: 4 mths and 3 wks	Randomized double blind placebo-controlled trial, crossover	YES (NGS)	PRO: ↑ LactImprovement of GI symptoms and anxiety compared to baseline, but without statistical significance	High dropout rates (10/13 completed the study) Small sample No TD control group
Inoue et al. (2019)	Japan	13 ASD 12 ♂ 1 ♀ age 4–9 yrs	PRE	Partially hydrolyzed guar gum (Taiyo Kagaku Co.Ltd., Mie, Japan) β-endogalactomannase produced by a strain of Asp. Niger	6 g/day Duration: 2–15 mths (median = 2)	Open-label, interventional study	YES (NGS)	Significant ↓ irritability after supplementation with partially hydrolyzed guar gum	Unblinded study No TD control group No PLA group Small sample
Kang et al. (2019)	U.S.	18 ASD with GI symptoms (moderate/severe) 16 ♂ 2 ♀ age 7–17 yrs	FMT	SHGM orally or rectally	Initial dose 2.5 × 10^12^ cells/day and maintenance dose 2.5 × 10^9^ cells/day for 7 or 8 weeks (+vancomycin + MoviPrep + Prilosec) Duration: 2-year follow-up	Open-label trial	YES (NGS)	Changes in gut microbiota lasted for 2 yrs, including significant ↑ in bacterial diversity and relative abundance of Bifid	ASD symptoms changes not reported Small sample, unblinded study No TD control group No PLA group
Liu et al. (2019)	China	39 ASD with PRO 41 ASD with PLA 80 ♂ 0 ♀ age 7–15 yrs	PRO	Lact plantarum PS128, 3 × 10^10^ CFU cp	1 cp/day × 1 mth	Randomized double blind placebo-controlled trial	YES (NGS)	↓ anxiety, hyperactivity and opposition/defiance behaviors; no change in the ASD symptoms	High dropout rates (9 out 80 subjects) Microbiota not analyzed No TD control group
Niu et al. (2019)	China	114 ASD (22 GI with PRO + ABA; 15 NGI with PRO + ABA; 28 ABA) ♂ ♀missing 40 TD age 3–8 yrs	PRO	3 Lact strains (bulgaricus, acidophilus, casei) + 3 Bifid strains (infantis, longum, bifidum)	6 g/day (36 billion CFU in total) + ABA training Duration: 1 mth	Open-label, two-arm, randomized trial	YES (NGS)	PRO + ABA vs only ABA: ↓ Total and subdomain ATEC scores; ↓ GI in 86.4% of 22 ASD GI with PRO + ABA	Small sample, unblinded study
Sanctuary et al. (2019)	U.S.	(20 ASD initially screened) 8 ASD with GI symptoms 7♂ 1 ♀age 2–11 yrs	PRE + PRO	Bifidobacterium infantis in combination with a bovine colostrum product (BCP) as a source of oligosaccharides	PRO 20 billion CFU/day, BCP 5.1–10.8 g/day 4 ASD with PRO + BCP 4 ASD with BCP 5 wks + 2 wks wash out + 5 wks	Randomized double blind trial, crossover	YES (NGS)	Combined treatment: some participants ↓ frequency of GI symptoms (++pain, diarrhea, stool consistency) and some atypical behaviors (++irritability, stereotypies, hypo/ hyperactivity) ↓ IL-13 and TNFα production in some participants	High dropout rates (8/20 completed the study) Lack of a control group with PLA and a PRO-only group No TD control group
Santocchi et al. (2020)	Italy	85 ASD (30 GI and 55 NGI) 71♂ 14 ♀ Average age 4.2 yrs	PRO	De Simone formulation-Vivomixx^®^ (1 Strept strain + 3 Bifid strains + 4 Lact strains)	2 packets/day (900 billions of bacteria) in the first mth and 1 packet/day (450 billions of bacteria) for the next 5 months	Randomized double blind placebo-controlled trial	NO	NGI PRO vs NGI PLA groups: ↓ ADOS GI PRO vs GI PLA groups: ↑ improvements in some GI symptoms, adaptive functioning and sensory profiles	High dropout rates (>GI group), 63/85 completed the study No information about microbiota No TD control group
Wang et al. (2020)	China	26 ASD (16 ASD with PRE + PRO; 10 ASD with PLA) 24♂ 2 ♀ age 3–9 yrs	PRE + PRO	4 PRO strains (Bifid infantis and lactis, Lact rhamnosus and paracasei) + fructooligosaccharide (FOS)	10^10^ CFU/pack/day 1, 2 or 3.6 mths	Randomized double blind placebo-controlled trial	YES (NGS)	↓ Total and subdomain ATEC scores compared to baseline ↓ Total 6-GSI score	Lack of a PRO-only group No TD control group
Mensi et al. (2021)	Italy	131 ASD 112 ♂ 19 ♀ Average age 86.1 ± 41.1 mths	PRO	Lact plantarum (105 ASD), OP (26 ASD)	Lact plantarum group: 3 × 10^10^ CFU if weight < 30 kg, 6 × 10^10^ CFU if weight > 30 kg OP group: prescribed PRO based on age, weight, and specific product Duration: 6 mths	Open-label trial	NO	↑ level of shared attention (54 ASD), ↓ stereotyped movements (43 ASD), ↑ communication skills (32 ASD) and ↑ personal autonomies (23 ASD) Higher improvements in Lact plantarum group No different improvements between GI and NGI subjects	Unblinded study Unbalanced number of subjects between Lact plantarum and OP groups Heterogeneous treatment in OP group Microbiota not analyzed No TD control group No PLA group
Li et al. (2021)	China	40 ASD (37 ♂ 3 ♀) age 8.03 ± 3.73 yrs 16 TD; age 7.13 ± 3.20 yrs	FMT	SHGM orally or rectally	Rectal route: dose of 2 × 10^14^ CFU, 50–100 mL per child, once a week. Oral route: dose of 2 × 10^14^ CFU, 8–16 capsules per child, once a week (+polyethylene glycol) Duration: 1 mth of FMT and 2 mths FU after the end of treatment	Open-label trial	YES (NGS)	↓ 35% reduction of GI symptoms at the end of treatment, lasting 8 wks after treatment, improvement of stool properties at the end of treatment compared to baseline, persisting 8 wks after FMT in ASD children. Mood, behavior, emotion, language and core ASD symptoms improved after FMT ↓ parents’ anxiety levels	Unblinded study No PLA group

Abbreviations (alphabetic order): ↑: increase, ↓: decrease, ♀: females, ♂: males, 6-GSI: six-gastrointestinal severity index, ABA: applied behavior analysis, ADI: autism diagnostic interview, ASD: autism spectrum disorder, Asp: Aspergillus, ATEC: autism treatment evaluation checklist, Bifid: Bifidobacterium, CARS: childhood autism rating scale, CFU: colonies forming units, CGI: clinical global impression, cp: capsule, FISH: fluorescence in situ hybridization, FMT: fecal microbiota transplantation, FU: follow-up, GI: gastrointestinal, g: grams, HC: healthy controls, Lact: Lactobacillus, mths: months, MTT: microbiota transfer therapy, NGS: next generation sequencing, NGI: not gastrointestinal, OP other probiotics, PLA: placebo, PRE: prebiotics, PRO: probiotics, SHGM: standardized human gut microbiota, Strept: Streptococcus, TD: typically developing children, TNFα: tumor necrosis factor α, VA: vitamin A, wks: weeks, yrs: years.

**Table 2:** Results of methodological quality assessment of randomized controlled trials.

First Author, Year	Randomization Process	Deviations from Intended Interventions	Missing Outcome Data	Measurement of the Outcome	Selection of the Reported Result	Overall Risk-of-Bias
Parracho, 2010	Low	High	High	Low	Low	High
Grimaldi, 2018	Low	Low	High	Low	Unclear	High
Arnold, 2019	Low	Low	Low	High	Low	High
Liu, 2019	Low	Low	Low	Low	Low	Low
Sanctuary, 2019	Low	Low	High	Low	Low	High
Santocchi, 2020	Low	Low	Low	Low	Low	Low
Wang, 2020	Low	High	High	Low	High	High

**Table 3:** Results of methodological quality assessment of nonrandomized studies.

	Pre-Intervention		At Intervention	Postintervention				
First Author, Year	Bias Due to Confounding	Bias in Selection of Participants into the Study	Bias in Classification of Interventions	Bias Due to Deviations from Intended Interventions	Bias Due to Missing Data	Bias in Measurement of Outcomes	Bias in Selection of the Reported Result	Overall Risk-of-Bias
Sandler, 2000	High	High	Low	High	High	Low	Low	High
Kaluzna-Czaplinska, 2012	Low	High	High	Low	Low	High	Low	High
West, 2013	High	High	Low	High	High	High	High	High
Kang, 2017 and 2019	High	High	Low	High	High	Low	High	High
Liu, 2017	Unclear	Low	Low	Low	High	Low	High	High
Guo, 2018	High	High	Low	High	Low	High	High	High
Shaaban, 2018	Low	Low	Low	High	Unclear	Low	High	High
Inoue, 2019	Low	Unclear	Unclear	High	Low	Low	High	High
Niu, 2019	Low	Unclear	High	High	High	Low	High	High
Mensi, 2021	High	Low	High	High	Low	High	High	High
Li, 2021	High	High	Low	Low	Low	Low	High	High

## Data Availability

The datasets used and/or analysed during the review are available from the corresponding author on reasonable request.
